# Deciphering the Biological Mechanisms Underlying the Genome-Wide Associations between Computerized Device Use and Psychiatric Disorders

**DOI:** 10.3390/jcm8122040

**Published:** 2019-11-21

**Authors:** Frank R Wendt, Carolina Muniz Carvalho, Gita A. Pathak, Joel Gelernter, Renato Polimanti

**Affiliations:** 1 Department of Psychiatry, Yale School of Medicine and VA CT Healthcare Center, West Haven, CT 06516, USA; 2 Universidade Federal de São Paulo (UNIFESP), São Paulo, SP 04021-001, Brazil; 3 Departments of Genetics and Neuroscience, Yale University School of Medicine, New Haven, CT 06510, USA

**Keywords:** *DRD2*, *FOXP2*, attention deficit hyperactivity disorder, schizophrenia, psychiatry, Mendelian randomization

## Abstract

Computerized device use (CDU) is societally ubiquitous but its effects on mental health are unknown. We performed genetic correlation, Mendelian randomization, and latent causal variable analyses to identify shared genetic mechanisms between psychiatric disorders (Psychiatric Genomics Consortium; 14,477 < N < 150,064) and CDU (UK Biobank; N = 361,194 individuals). Using linkage disequilibrium score regression, we detected strong genetic correlations between “*weekly usage of mobile phone in last 3 months”* (*PhoneUse*) vs. attention deficit hyperactivity disorder (ADHD; *r*_g_ = 0.425, *p* = 4.59 × 10^−29^) and “*plays computer games”* (*CompGaming*) vs. schizophrenia (SCZ; *r*_g_ = −0.271, *p* = 7.16 × 10^−26^). Focusing on these correlations, we used two sample MRs to detect the causal relationships between trait pairs by treating single nucleotide polymorphisms as non-modifiable risk factors underlying both phenotypes. Significant bidirectional associations were detected (*PhoneUse*→ADHD *β* = 0.132, *p* = 1.89 × 10^−4^ and ADHD→*PhoneUse β* = 0.084, *p* = 2.86 × 10^−10^; *CompGaming*→SCZ *β* = −0.02, *p* = 6.46 × 10^−25^ and *CompGaming*→SCZ *β* = −0.194, *p* = 0.005) and the latent causal variable analyses did not support a causal relationship independent of the genetic correlations between these traits. This suggests that molecular pathways contribute to the genetic overlap between these traits. Dopamine transport enrichment (Gene Ontology:0015872, *p_SCZvsCompGaming_* = 2.74 × 10^−10^) and *DRD2* association (*p*_SCZ_ = 7.94 × 10^−8^; *p_CompGaming_* = 3.98 × 10^−25^) were detected in SCZ and *CompGaming* and support their negative correlative relationship. *FOXP2* was significantly associated with ADHD (*p* = 9.32 × 10^−7^) and *PhoneUse* (*p* = 9.00 × 10^−11^) with effect directions concordant with their positive genetic correlation. Our study demonstrates that epidemiological associations between psychiatric disorders and CDUs are due, in part, to the molecular mechanisms shared between them rather than a causal relationship. Our findings imply that biological mechanisms underlying CDU contribute to the psychiatric phenotype manifestation.

## 1. Introduction

The information revolution has contributed to humans’ reliance on technology to store and transmit ideas to the point where computerized device use (CDU) is societally ubiquitous [1]. While these developments are generally beneficial, there is growing discussion about potential harms as well [2,3,4,5,6,7]. A relationship between personal CDU (e.g., cellular phones, video/computer games, desktop/laptop computers) and psychiatric traits (i.e., inattention [7,8]), psychiatric disorders (attention deficit hyperactivity disorder (ADHD) [2,9], anxiety disorders [10], and schizophrenia (SCZ)) has been established in epidemiological studies. While these relationships are often explored as potentially detrimental to human health [2,7,10], there are data suggesting that CDUs may also be beneficial to certain psychiatric conditions (e.g., video games seem to have a therapeutic effect on SCZ [11]). Causality between CDUs and psychiatric disorders has been difficult to determine due to combined effects of psychiatric disorder heterogeneity and sample size constraints [12] (e.g., disorder heterogeneity may add noise to studies with goals to detect causality)—and importantly, the inability to use conventional designs in a single-ascertainment context to draw such conclusions. Genetic information from large-scale genome-wide association studies (GWAS) can be used to combat these limitations and detect genetic overlap, causal relationships, and molecular pathways shared between complex traits [13,14]. Elucidating the mechanisms linking these traits will lead to improved understanding of the possibly long-term effects of CDUs and may conceivably contribute to developing nonpharmacological therapies for various disorders and provide biological evidence supporting the use of these therapies in combination with pharmacological intervention. 

We hypothesize that genetic information underlying CDU and psychiatric disorders explains the epidemiological relationships between them. We tested this hypothesis by investigating causal relationships, genetic overlap, and shared molecular mechanisms linking these traits using GWAS summary association data (Table 1) generated by the Psychiatric Genomics Consortium (PGC) [15] and the UK Biobank (UKB) [16].

## 2. Materials and Methods

### 2.1. Genetic Data

Summary statistics of five CDU measures and eight psychiatric disorders (i.e., ADHD [17], AD [18], AN [19], ASD [20], BIP [21], MDD [22], PTSD [23] and SCZ [24]) were obtained from PGC and the UKB) (Table 1). The UKB represents a collection of deeply phenotyped individuals from the UK in the adult age range (approximately 40–60 years of age). We used five CDU phenotypes from this biobank: *For approximately how many years have you been using a mobile phone at least once per week to make or receive calls?* (UKB Field ID: 1110), *Over the last three months, on average how much time per week did you spend making or receiving phone calls on a mobile phone?* (*PhoneUse*; UKB Field ID: 1120), *Hands-free device/speakerphone use with mobile phone in the last three months* (UKB Field ID: 1130), *Difference in mobile phone use compared to two years previously* (UKB Field ID: 1140), and *Plays computer games* (*CompGaming*; UKB Field ID: 2237). All phenotypes have been represented in the biobank as ordinal measures of CDU behavior and the specific categorical distributions for UKB participants of all ancestries are shown in Table 1. Due to the limited representation of individuals of non-European descent in these previous studies, all analyses were restricted to GWAS summary association data for individuals of European ancestry. The association analysis for all phenotypes was conducted using appropriate regression models available in Hail (available at https://github.com/hail-is/hail) including the first 20 ancestry principal components, sex, age, age^2^, sex × age, and sex × age^2^ as covariates. Additional details regarding quality control criteria and GWAS methods for the UKB and PGC are available at https://github.com/Nealelab/UK_Biobank_GWAS/tree/master/imputed-v2-gwas and https://www.med.unc.edu/pgc/results-and-downloads, respectively. Details regarding the original GWAS analyses and findings from PGC are described in previous articles [17,18,19,20,21,22,23,24]. GWAS for CDU phenotypes in the UKB use raw ascertained data; however, some of the continuous phenotypes used for subsequence phenome-wide genetic correlations are available in raw ascertainment form and inverse rank normalized form (i.e., they are transformed to fit a normal distribution). For these continuous phenotypes, we used the normalized GWAS summary data only.

### 2.2. Genetic Correlation

GWAS summary association data related to CDUs (Table 1) and psychiatric disorders were used to calculate heritability and genetic correlation using the linkage disequilibrium score regression (LDSC) method [25,26]. Bonferroni correction was performed using the p.adjust function in R studio. Subsequent genetic correlations were evaluated using GWAS summary statistics for the remaining UKB traits (4085 for both sexes combined, 3291 for females only, and 3148 for males only). Note that the methods used to identify suitable genetic instruments (e.g., output from PRSice v1.25) and evaluate causality between traits (e.g., two-sample Mendelian randomization) are sensitive to sample overlap [27]. To avoid possible sample overlap, versions of PGC GWAS summary data known to exclude UKB samples were used for this study: MDD and SCZ from Wray et al. [22] and the PGC Schizophrenia Working Group et al. [24], respectively, rather than Howard et al. [28] and Pardinas et al. [29], respectively, which either knowingly or potentially overlap with UKB participants (i.e., Pardinas et al. includes individuals from the CLOZUK study of UK participants).

### 2.3. Genetic Instrument Selection

PRSice v1.25 [30] was used to identify a significance threshold for inclusion of variants in the genetic instrument in order to maximally explain the cross-phenotype variance between CDU and psychiatric disorders. Variants with association *p*-values less than this significance threshold were included in the genetic instruments for Mendelian randomization. For this SNP-selection process, we used stringent clumping criteria (clump_kb_ = 10,000 and clump_r2_ = 0.001) to minimize correlation among genetic instruments, which may bias downstream causal estimates between phenotypes. The region encoding the major histocompatibility complex was removed from this process due to its complex linkage disequilibrium structure. Note that (1) outputs from PRSice v1.25 were not interpreted for predictability of an outcome trait using genetic data from an exposure trait, as is the typical application of PRSice v1.25 and (2) Trait 1 predicts Trait 2 differently than Trait 2 predicts Trait 1 and as such, the number and identity of the genetic instruments identified through these analyses may be different for each trait pair and each analysis direction for any given trait pair.

### 2.4. Mendelian Randomization

The causality between CDU and psychiatric disorders was evaluated using two-sample Mendelian randomization (MR) with genetic instruments included based on the best-fit *p*-value threshold from PRSice v1.25 (ranging from 1.0 to 5 × 10^−8^, see Genetic Instrument Selection). This approach has proven well-powered for overcoming the combination of high polygenicity and relatively few, and weakly associated, risk loci contributing to psychiatric disorders relative to a more traditional MR approach strictly using genome-wide significant loci in the genetic instruments [14,31,32,33,34]. Using the best-fit PRSice v1.25 model to select genetic instruments may exacerbate certain MR sensitives (e.g., many weak genetic instruments included in the analysis), so multiple methods were used here to verify the stability of causal estimates across methods, mindfully remove outliers, and evaluate estimate sensitivity. These included causal estimates based on mean [35], median [36], and mode [37] with various adjustments to accommodate weak instruments [38] and the presence of horizontal pleiotropy (i.e., when genetic variants associate with the outcome independently of the exposure) [39,40]. Appropriate sensitivity tests were used to evaluate the presence of pleiotropic effects (MR-Egger, MR robust adjusted profile score (RAPS) over dispersion and loss-of-function [38], and MR pleiotropy residual sum and outlier (PRESSO [40]), heterogeneity, and the pervasive effect of outlier variants in the genetic instrument (leave-one-out). The strict quality control employed here combined with the large samples sizes for each GWAS used reduced possible violations of MR assumptions while selecting genetic instruments to test for causality between trait pairs [32]. Two sample MR tests were conducted using the R package TwoSampleMR [41]. It should be noted that our trait pairs of interest indeed demonstrated effects of horizontal pleiotropy and outliers within the genetic instruments. We removed these variants and only report results devoid of horizontal pleiotropy and variants with outlier effects as determined by several sensitivity methods.

Multivariable MR was performed using the MendelianRandomization R package [42,43] and genetic instruments contributing to the best-fit PRSice v1.25 model for each exposure trait.

The latent causal variable (LCV) model [44] was used to estimate the genetic causality proportion (gĉp) between traits using *z*-score converted per-variant effects and regression weights for genome-wide summary statistics. LCV uses genome-wide data (rather than select genetic instruments) to evaluate whether causal estimates (i.e., the genetic liability of the exposure has a causative effect on the outcome; e.g., those derived from MR) are independent of the genetic correlations (i.e., the association of per-SNP effect estimates) between traits.

It should be noted that MR and LCV causal estimates can be interpreted as Trait 1 (estimated by *β*) causes, or protects against, a portion of the risk for exhibiting/developing Trait 2.

### 2.5. Enrichment Analyses

GWAS summary statistics were annotated for (1) physical proximity to genes (window size = 10 kb) and (2) enrichment of molecular pathways and gene ontology (GO) annotations (*n* = 10,651) using Multi-marker Analysis of GenoMic Annotation (MAGMA v1.06) [45], implemented in FUnctional Mapping and Annotation (FUMA) v1.3.3c [46] with the following parameters: genome-wide significance *p* < 5 × 10^−8^, minor allele frequency ≥ 0.01, and LD blocks merged at <250 kb for LD *r*^2^ ≥ 0.6 with lead variant. *Z*-tests were used to determine differences in gene set enrichment and genetic correlations. The SNPs underlying each trait were mapped to genes within a 10 kb physical proximity and were assessed for enrichment of ~17,000 gene sets.

## 3. Results

### 3.1. Genetic Correlation between Computerized Device Use and Psychiatric Disorders

**Figure 1:**
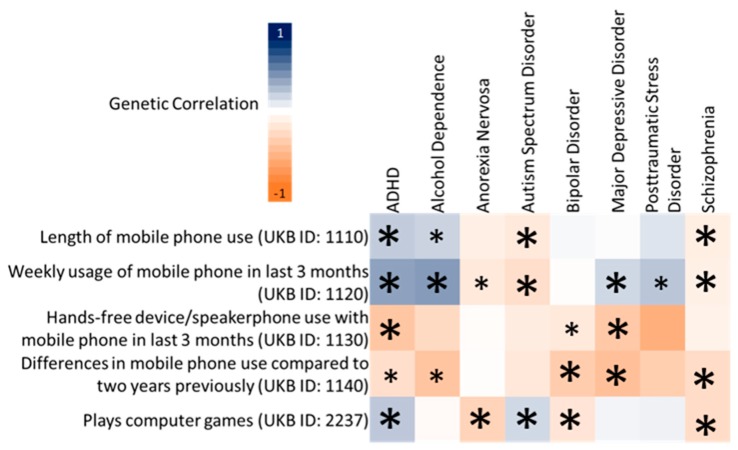
Linkage disequilibrium score regression genetic correlations for computerized device use and psychiatric disorder (attention deficit hyperactivity disorder (ADHD), alcohol dependence, autism spectrum disorder, bipolar disorder, anorexia nervosa, major depressive disorder, post-traumatic stress disorder, and schizophrenia) pairs. Large asterisks indicate genetic correlations surviving multiple testing correction (*p* = 1.25 × 10^−3^), while small asterisks indicate nominally significant genetic correlations. Genetic correlations significant after Bonferroni correction are provided in Table S1.

All CDUs were significantly heritable with *h*^2^ *z*-scores greater than 4 indicating appropriate power for LDSC (Table 1). A total of 18 significant genetic correlations were observed between CDU and psychiatric disorders after Bonferroni correction (*p* < 1.25 × 10^−3^; Figure 1 and Table S1). The UKB CDU traits *PhoneUse* and *CompGaming* were strongly genetically correlated with several psychiatric disorders (8/18 significant genetic correlations involved these CDUs). MR studies using psychiatric phenotypes are prone to detecting relatively small effect estimates and therefore often meet levels of nominal significance [14,47,48,49]. To avoid the potential for over-correcting *p*-values from MR analyses, we selected the two trait pairs with highest genetic correlation *p*−values: (1) ADHD versus *PhoneUse*; (*r*_g_ = 0.425, *p* = 4.59 × 10^−29^) and (2) schizophrenia versus *CompGaming* (*r*_g_ = −0.271, *p* = 7.16 × 10^−26^). 

Sex−stratified genetic correlation (available for ADHD and PTSD; Table S1) was substantially greater between ADHD and *PhoneUse* in females (*r*_g_ = 0.710, *p* = 5.91 × 10^−31^) than in males (*r*_g_ = 0.322, *p* = 9.51 × 10^−7^; difference compared to females: *z* = 3.25, *p* = 0.001).

### 3.2. Mendelian Randomization

PRSice v1.25 was used to select genetic instruments with which to perform MR analyses (Additional Data Tables 1–3). The PRSice v1.25 model of CDU on psychiatric disorders was consistently more powerful than the reverse relationship so MR tests were performed assuming this direction as the main hypothesis (see MR methods). Unless otherwise noted, the inverse-variance weighted (IVW) [36] causal estimate is discussed herein while all causal estimates are provided in Table S2. Specifically, we applied random-effect IVW, which is less affected by heterogeneity among the variants included in the genetic instrument than a fixed-effect model. The causal effects (*β*; Figure S1), not affected by heterogeneity and horizontal pleiotropy (Table S2), were bidirectional with CDUs having greater effects on psychiatric disorders (*PhoneUse*→ADHD *β*_IVW_ = 0.132, *p*_IVW_ = 1.89 × 10^−4^; *CompGaming*→SCZ *β*_IVW_ = −0.194, *p*_IVW_ = 0.005) than the reverse relationship (ADHD→*PhoneUse β*_IVW_ = 0.084, *p*_IVW_ = 2.86 × 10^−10^; SCZ→*CompGaming β*_IVW_ = −0.020, *p*_IVW_ = 6.46 × 10^−25^). These effects were significantly stronger for CDU→PsychiatricDisorder than the reverse direction (*z*_ADHD_*PhoneUse*_ = 2.28, *p* = 0.023; *z*_SCZ_*CompGaming*_ = −3.56, *p* = 3.71 × 10^−4^). When stratified by sex, the female *PhoneUse*→ADHD relationship was nearly 4-fold greater than that in males (*β*_IVW_female_ = 0.287, *p*_IVW_female_ = 1.09 × 10^−7^; *β*_IVW_male_ = 0.080, *p*_IVW_male_ = 0.019).

Multivariable MR was performed to verify the causal estimates of the SCZ→*CompGaming* and ADHD→*PhoneUse* relationships given the genetic correlations of other psychiatric disorders with the same CDU. When adjusted for ADHD diagnosis, only the negative causal estimate between ASD_ADHD_→*PhoneUse* remained significant (original two-sample *β*_IVW_ = −0.019, *p*_IVW_ = 1.45 × 10^−4^; multivariable *β*_IVW_ = −0.044, *p*_IVW_ = 2.40 × 10^−8^; Figures S2–S3 and Tables S2–S3).

Full GWAS summary data for the relationship between (1) ADHD and *PhoneUse*, (2) SCZ and CompGaming, and (3) ASD and *PhoneUse* (selected due to multivariable MR significance detected above) were used to estimate the genetic causality proportion (gĉp) using regression weights via the LCV model (Table S4). LCV results for SCZ and *CompGaming* indicated that the datasets investigated are able to detect a causal relationship. We detected that the genetic risk for SCZ explains 8.1% of the genetic risk for *CompGaming* but this partial causal observation was not significant (gĉp *p*-value = 0.65). Partial causality estimates between ADHD, ASD, and *PhoneUse* were also not significant, so we concluded that the causal estimates between CDU traits and psychiatric disorders, as detected above with MR approaches, were not independent of the genetic correlations between them (i.e., strong genetic correlation between CDU and psychiatric disorders likely contributes to significant causal estimates detected with MR). Sex-stratified heritabilities were insufficient to reliably estimate the gĉp between trait pairs.

The combination of (1) bidirectional causal estimates between trait pairs using MR and (2) lack of genome-wide causal estimates that are independent of genetic correlations (i.e., observed genetic correlations did not indicate causal relationships) suggest that the trait pairs tested share underlying biological features (e.g., shared molecular mechanisms and/or causal relationships with an unobserved mediator) rather than causal relationships [50]. These shared mechanisms and putatively mediating phenotypes are explored below using three approaches: (1) gene overlap, (2) gene set enrichment overlap, and (3) genetic correlate overlap.

### 3.3. Genetic Similarities and Differences between Computerized Device Use and Psychiatric Disorders

#### 3.3.1. Schizophrenia and CompGaming

**Figure 2:**
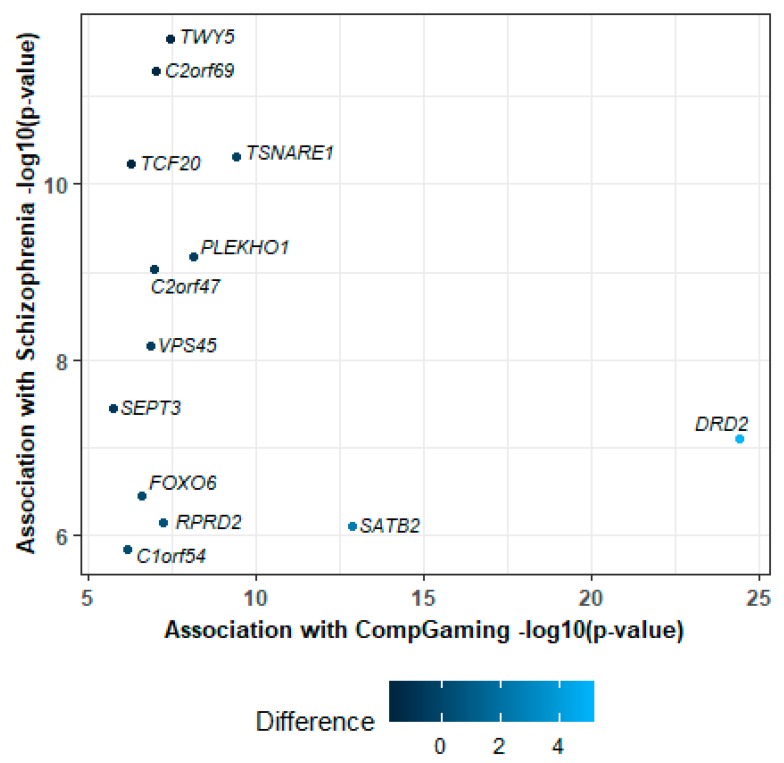
Genes significantly associated with both schizophrenia (SCZ) and UKB Field ID 2237 “*plays computer games*” (*CompGaming*) after Bonferroni correction (*p* < 2.64 × 10^−6^). The difference in per-gene effects (*z-*score*_CompGaming_* minus *z*-score_SCZ_) on each trait is color-coded.

SCZ and *CompGaming* have a negative genetic correlation suggesting shared genetic architecture, perhaps with opposing effects, contributing to phenotype. Using a SNP-based approach, one variant (rs62512616) met genome-wide significance in both SCZ (36,989 cases and 113,075 controls; 2.30 × 10^−9^; LD *r*^2^ < 0.1; Table S5) and *CompGaming* (301,157 subjects; *p* = 3.01 × 10^−8^; (LD *r*^2^ < 0.1; Table S6) GWASs; it maps to the intronic region of *t-*SNARE domain containing 1 (*TSNARE1*) and has opposite effects in SCZ (beta = 0.025, SE = 0.011 and *CompGaming* (beta = −0.007, SE = 0.001). Using a gene-annotation approach, significant variants mapped to a total of 13 genes; one (rs2514218) and three (rs2734837, rs4648319, and rs4936271) lead variants from the SCZ and *CompGaming* GWAS, respectively, mapped to the dopamine receptor D2 (*DRD2*) gene. In addition to *DRD2* and *TSNARE1*, 11 other genes met genome-wide significance for both traits in a gene-based GWAS (*p* < 2.64 × 10^−6^; Figure 2). All 13 genes had positive *z*-score converted effects in SCZ and *CompGaming* (Figure 2).

**Figure 3:**
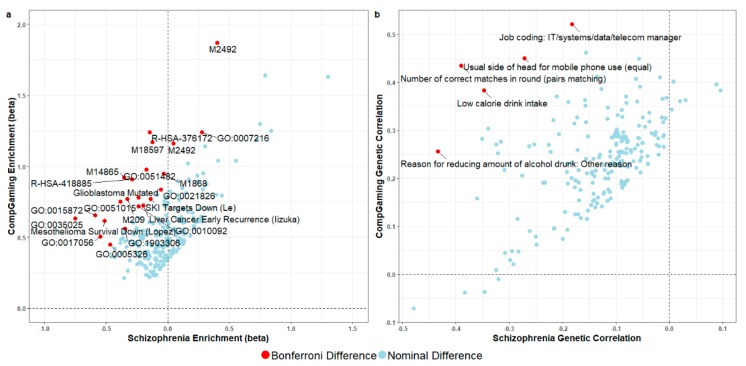
Scatterplots showing gene set enrichment (**a**; *N* = 221 gene sets) and linkage disequilibrium score regression (**b**; *N* = 224 traits) results for gene sets and UK Biobank traits with nominally significant differences between schizophrenia and *CompGaming* (UKB Field ID 2237 “*plays computer games*”). Gene sets are labeled with gene ontology (GO), reactome (R), systematic identifiers (M), or specific study identifiers (last name of first author); the top five most significant differences in genetic correlation are labeled. Detailed results for gene set enrichment and genetic correlations surviving multiple testing correction are provided in Tables S7–S10.

Twenty-two molecular pathways showed differential enrichments in line with the negative association between SCZ and *CompGaming* (*p* < 4.69 × 10^−6^; Figure 3 and Table S7). These differential mechanisms include gene sets related to the stress stimulus response (e.g., systematic name M2492: MAPK11 (*p* = 1.02 × 10^−13^) and MAPK14 targets (*p* = 1.25 × 10^−8^); and the PID p38-gamma and p38-delta pathway (*p* = 1.96 × 10^−8^) and synapse structure, plasticity, function, and neurotransmitter signaling (e.g., reactome Down syndrome cell adhesion molecule interactions (R-HSA-376172) *p* = 2.17 × 10^−13^; GO:0015872 dopamine transport *p* = 2.74 × 10^−10^; GO:0007186 G-protein coupled glutamate receptor signaling pathway *p* = 7.22 × 10^−7^; and GO:0006836 neurotransmitter transporter activity *p* = 2.40 × 10^−6^). The six and seven gene set enrichments surviving Bonferroni correction in SCZ and *CompGaming*, respectively, are provided in Tables S8 and S9, respectively.

To uncover the putative mediator phenotype between the SCZ and *CompGaming* association, genetic correlation was calculated between 4085 phenotypic traits and SCZ/*CompGaming* (Figure 3 and Table S10). Five traits had significant differences between SCZ and *CompGaming* in line with the negative association between these traits (Table S10). Three of these significantly different traits also were significantly associated with SCZ and *CompGaming* after Bonferroni correction (*p* < 1.22 × 10^−5^): “*number of correct matches in round (pairs matching)*” (UKB Field ID 398: *rg*_SCZ_ = −0.390, *p*_SCZ_ = 4.33 × 10^−27^; *rg_CompGaming_* = 0.435, *p_CompGaming_* = 6.69 × 10^−25^), “*usual side of head for mobile phone use (equal)*” (UKB Field ID 1150_3: *rg*_SCZ_ = −0.272, *p*_SCZ_ = 4.51 × 10^−9^; *rg_CompGaming_* = 0.450, *p_CompGaming_* = 2.49 × 10^−17^), and “*reason for reducing amount of alcohol drunk: other reason*” (UKB Field ID 2664_5: *rg*_SCZ_ = −0.434, *p*_SCZ_ = 6.35 × 10^−27^; *rg_CompGaming_* = 0.256, *p_CompGaming_* = 1.09 × 10^−11^). Two hundred and sixty-three and 348 UKB traits were independently significantly associated with SCZ and *CompGaming*, respectively, after Bonferroni correction (*p* < 1.14 × 10^−5^; Figure 3 and Table S10).

#### 3.3.2. ADHD and *PhoneUse*

ADHD and *PhoneUse* showed a genetic correlation that was stronger in females than males. The shared genetic architecture of these traits was investigated using genome-wide data considering both sexes combined (ADHD: 19,099 cases and 34,194 controls; *PhoneUse*: 356,618 subjects), females (ADHD: 4,945 cases and 16,246 controls; *PhoneUse*: 191,522 subjects), and males (ADHD: 14,154 cases and 17,948 controls; *PhoneUse*: 165,096 subjects). Using a SNP-based approach, there was no overlap in genomic risk loci or individual significant variants between ADHD (LD *r*^2^ < 0.1; Table S11) and *PhoneUse* (LD *r*^2^ < 0.1; Table S12) GWAS; however, individual significant loci from both GWAS map to genes involved in fear recognition/consolidation (sortilin related VPS10 domain containing receptor 3 (*SORCS3*)) in ADHD [50] and hypocretin receptor 2 (*HCRTR2*) [51] in *PhoneUse*) and language/speech development/impairment (Semaphorin 6D (*SEMA6D*) in ADHD [52] and forkhead box transcription factor (*FOXP2*) in ADHD and *PhoneUse* [53]). *FOXP2* was the only gene-based genome-wide significant result in both ADHD (*p* = 9.32 × 10^−7^) and *PhoneUse* (*p* = 9.00 × 10^−11^). Conversely, *FOXP2* (*p* = 0.084) was not significant in the ASD GWAS (18,382 cases and 27,969 controls), an observation in line with the independent associations of ADHD and ASD with *PhoneUse* (Tables S1 and S13). 

The GWAS for ADHD females and *PhoneUse* males were insufficiently powered to identify genome-wide significant variants so these data were explored using a suggestive threshold of *p* < 1 × 10^−5^ (LD *r*^2^ < 0.1; Tables S14–S17). There were no significant overlapping variants or genes between ADHD and *PhoneUse* after Bonferroni correction in the sex-stratified cohorts.

Ninety-one, 29, and 39 gene sets were enriched in both ADHD and *PhoneUse* for both sexes, males, and females, respectively. None of these gene sets survived multiple testing correction in both GWAS (Tables S18–S20).

**Figure 4:**
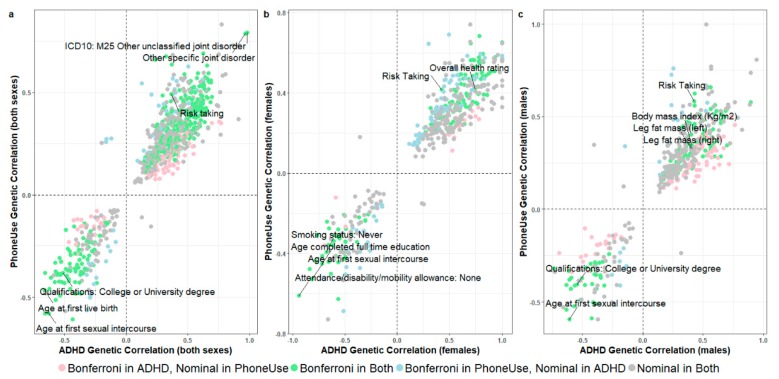
Scatterplots of genetic correlation results for attention deficit hyperactivity disorder (ADHD) and *PhoneUse*, for both sexes combined (**a**; *N* = 841 traits), females only (**b**; *N* = 478 traits), and males only (**c**; *N* = 435 traits). Labeled traits are among the top ten most significant genetic correlations in ADHD and *PhoneUse* (UKB Field ID 1120 “*weekly usage of mobile phone in last three months*”). Detailed genetic correlation results surviving multiple testing correction are provided in Tables S21–S23.

A total of 551 and 491 significant genetic correlations were detected for ADHD and *PhoneUse*, respectively. Three hundred and sixty-nine of these were significantly correlated with both traits after multiple testing correction (*p* < 1.22 × 10^−5^; Figure 4 and Table S21). Among the top ten traits correlated with ADHD and *PhoneUse*, three traits were shared: “*age at first sexual intercourse*” (UKB Field ID 2139: *rg*_ADHD_ = −0.623, *p*_ADHD_ = 2.17 × 10^−128^*; rg_PhoneUse_* = −0.579, *p_PhoneUse_* = 6.24 × 10^−132^), “*smoking status: never*” (UKB Field ID 20116_0; *rg*_ADHD_ = −0.523, *p*_ADHD_ = 1.91 × 10^−79^; *rg_PhoneUse_* = −0.386, *p_PhoneUse_* = 6.93 × 10^−56^), and “*qualifications: college or university degree*” (UKB Field ID 6138_1: *rg*_ADHD_ = −0.510, *p*_ADHD_ = 7.52 × 10^−74^; *rg_PhoneUse_* = −0.380, *p_PhoneUse_* = 1.70 × 10^−58^). These genetic correlations were replicated in females and males (Figure 3 and Tables S22 and S23), with “*age at first sexual intercourse*” as the most significant genetic correlation with ADHD and *PhoneUse* in the sex-stratified cohorts (UKB Field ID 2139: rg_ADHD_Females_ = −0.815, *p*_ADHD_Females_ = 3.25 × 10^−22^; *rg_PhoneUse_*__Females_ = −0.529, *p_PhoneUse_*__Females_ = 1.31 × 10^−64^; *rg*_ADHD_Males_ = −0.610, *p*_ADHD_Males_ = 6.56x10^−69^; r*g_PhoneUse_*__Males_ = −0.592, *p_PhoneUse_*__Males_ = 5.03 × 10^−57^). Differential gene set enrichments and genetic correlations between ASD and *PhoneUse* that survived multiple testing correction were observed but did not represent any singular molecular mechanism as was observed for dopamine enrichment between SCZ and *CompGaming* (Tables S24 and S25). 

## 4. Discussion

We have reported a high degree of genetic correlation between CDU traits and psychiatric disorders. The relatively large number of genetic correlations surviving multiple testing correction highlights the pleiotropic nature of genetic risk for these phenotypes. We demonstrated particularly strong genetic correlations between ADHD and SCZ with CDU, which survive multiple testing correction; however, ASD and major depression (MDD) also demonstrate several significant genetic correlations. We hypothesize that the overlap between the CDU traits genetically correlated with ADHD and ASD are likely due to the high genetic correlation between ADHD and ASD. The observations involving MDD were relatively weak in magnitude but had a very different correlated architecture than those of ADHD, ASD, and SCZ, which warrants additional future study.

Among the trait pairs tested for causal inference, we detected bidirectional causality using MR methods and the LCV analyses did not support a causal relationship independent of the genetic correlations between these traits. We therefore focused our analyses on the underlying biology that may be responsible for strong genetic correlations between CDU and psychiatric disorders. Specifically, we investigated the two strongest genetic correlations: (1) SCZ and *CompGaming* and (2) ADHD and *PhoneUse*. The genetic overlap detected between SCZ and *CompGaming* suggests that these phenotypes share underlying molecular mechanisms, which contribute to the two phenotypes. For example, enrichment of dopamine transport was detected with several analytic procedures (e.g., gene based, gene sets, and overlapping genetic correlates). Gene ontologies were significantly enriched in SCZ and *CompGaming* but had opposite directions of the effect, which suggests that genes contributing to dopamine transport work in opposing ways to produce the schizophrenia and computer gaming phenotypes. Gene-based GWAS analyses implicated *DRD2* and *TSNARE1* in the SCZ and *CompGaming* protective relationship. Our discussion focuses on this repeated detecting of dopamine processes. Genetic variation in *DRD2* and imbalance of the dopamine transport system in various brain regions are readily observed in SCZ patients [24] and most antipsychotic drugs are antagonists of D_2_ dopamine receptors (D_2_R), the protein product of *DRD2* [24]. Studies have shown that video and computer game playing increases release of dopamine in the striatum and this relationship is observed in our findings of enrichment of dopaminergic systems [54]. SCZ patients and healthy computer game users both exhibit increased dopamine levels in various brain regions suggesting that dopamine concentration per se, as opposed to alterations in its regulation, is an unlikely contributor to the SCZ-*CompGaming* negative relationship. TSNARE1 has recently shown synergistic effects with other putative SCZ risk loci but interactions between TSNARE1 and DRD2 remains unknown. Based on the findings of this study and the current literature, in SCZ patients lacking a secondary addiction diagnosis, the pairs-matching component of video and/or computer game playing may increase D_2_R density (perhaps through synergistic TSNARE1 mechanisms) in specific brain regions, thereby increasing grey matter density in the region over time and providing protective effects against SCZ symptoms.

We observed strong evidence of genetic overlap between *PhoneUse* and ADHD with sex-stratified differences. *FOXP2* was the only gene overlapping the ADHD and *PhoneUse* GWASs. The *FOXP2* locus encodes a brain-expressed transcription factor strongly implicated in the human ability to communicate via complex speech [53]. Additionally, we uncovered multiple genetically correlated traits shared between ADHD and *PhoneUse* in the combined and sex-stratified cohorts, including “*age at first sexual intercourse*” and “*risk taking*.” While these traits were detected by the PGC ADHD GWAS of both sexes combined [17], the connection to *PhoneUse* is perhaps best explored in the sex-stratified cohorts. The genetic correlation between males and females with ADHD is quite high, initially suggesting that the same set of common variants may contribute to ADHD in both sexes; however, this difference has previously been attributed to differences in ADHD prevalence between sexes [55]. Because ADHD prevalence and comorbid conditions differ between sexes, we considered mechanistic differences, which may contribute to higher correlation and causal inference in females relative to males.

Sex hormones are implicated in various aspects of human speech, including pitch, overall voice profile, and vocal structural anatomy, many of which are influenced by androgen/testosterone expression differences between males and females [56]. In rates, *FOXP2* expression is dampened by androgen/testosterone expression and androgen receptor density is modulated by testosterone [57]. *FOXP2* also is expressed in brain regions in which androgen receptor density is highly responsive to androgen/testosterone [57]. The presence of “*age at first sexual intercourse*” and “*risk taking*” as genetic correlates herein and in additional studies also complements testosterone or related mechanisms with *PhoneUse* and ADHD [58]. These data support the possibility that sex hormone expression differences in males and females contribute to *FOXP2* expression differences, resulting in observations of stronger genetic correlation estimates between ADHD and *PhoneUse* in females. 

Our study has several limitations. First, although sample sizes for large-scale genetic studies are capable of reducing the effects of psychiatric disorder heterogeneity on analogous epidemiological studies (e.g., randomized controlled trails), GWAS are not devoid of the effects of diagnostic heterogeneity that may exist in the GWAS used for this study. A potential consequence of this heterogeneity may indeed be sufficient genome-wide noise to mask the strength of causal relationships, such as those not detected with the LCV approach using genome-wide variants rather than targeted variants. This may be especially true for use of computerized devices, which have substantial ascertainment differences between older and younger individuals. Therefore, our lack of detection of causal relationships between CDU and psychiatric disorders may be restricted to adults. It remains undetermined if similar observations are true in youths who have more ubiquitous CDU behavior across their lifetime. Second, the use of reduced genome-wide significance thresholds for selecting MR instrumental variables is an unconventional procedure. However, when accompanied by rigorous sensitivity testing of SNPs with effect size outliers, tests for horizontal pleiotropy, and causal inference tests specifically designed to overcome weak instruments, the approach employed here adheres to MR assumptions [59]. Although sample size is tightly related to the utility of weak instrumental variables, it is possible that those variables included here bias causal inferences towards the confounding association. We supplemented our MR causal inferences with LCV testing and indeed cannot distinguish genetic correlation from causation. As GWAS sample sizes continue to increase and improve upon the power of genetic instruments and their association with exposure phenotypes, causal inferences between CDU and psychiatric disorders may become more stable [31,32]. Third, we tested multivariable MR including a second psychiatric disorder that was genetically correlated with the CDU of interest (e.g., we included ASD in the causal relationship between ADHD and *PhoneUse*). We did not test other potential mediating variables. This means that, although it seems unlikely due to the several sensitivity analyses conducted, an unaccounted mediator may have a causal effect on both psychiatric disorders and CDU phenotypes, generating the genetic correlation observed. Lastly, although we have corrected for multiple testing, selection of an appropriate *p*-value threshold with which to determine a result significant is perhaps confounded by lack of independence between genetic correlations and MR. With respect to our study, a Bonferroni threshold *(p* < 0.001) would not change our interpretation of the results presented. Future MR studies will need to address suitable multiple testing correction strategies given the high number of tests performed (i.e., bidirectional MR, multivariable MR, several MR testing methods, and combinations of these testing strategies).

## 5. Conclusions

Our findings demonstrate that CDUs have strong genetic overlap with psychiatric disorders (Figure S4), though these results do not support a unidirectional causal relationship between CDU and psychiatric disorders. These genetic relationships point to potential mechanistic insights into epidemiological correlations between CDUs and psychiatric disorders to identify prospective pharmacological and nonpharmacological therapeutic targets for psychiatric disorders.

## Figures and Tables

**Table 1:** General information for computerized device UK Biobank (UKB) traits and Psychiatric Genomes Consortium (PGC) disorders. Note that data distributions are based on the entire UKB.

Trait	UKB Questionnaire Entry	Abbreviation	Sample Size	Heritability % (se)	Cohort	Phenotype Description/Reference to Original Work
Length of mobile phone use (UKB ID: 1110)	*For approximately how many years have you been using a mobile phone at least once per week to make or receive calls?*	*PhoneLength*	356,618	5.35 (0.26)	UKB	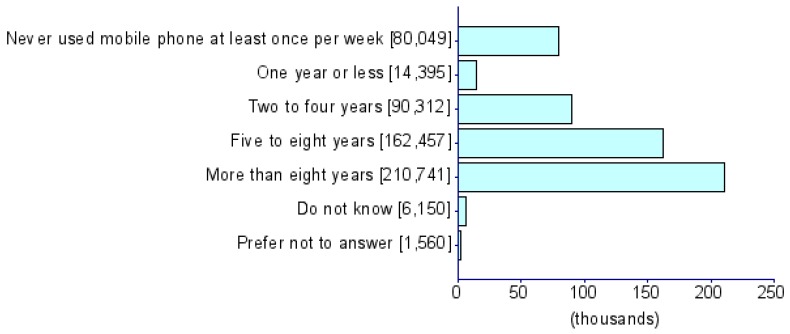
Weekly usage of mobile phone in last 3 months (UKB ID: 1120)	*Over the last three months, on average how much time per week did you spend making or receiving calls on a mobile phone?*	*PhoneUse*	301,157	4.82 (0.25)	UKB	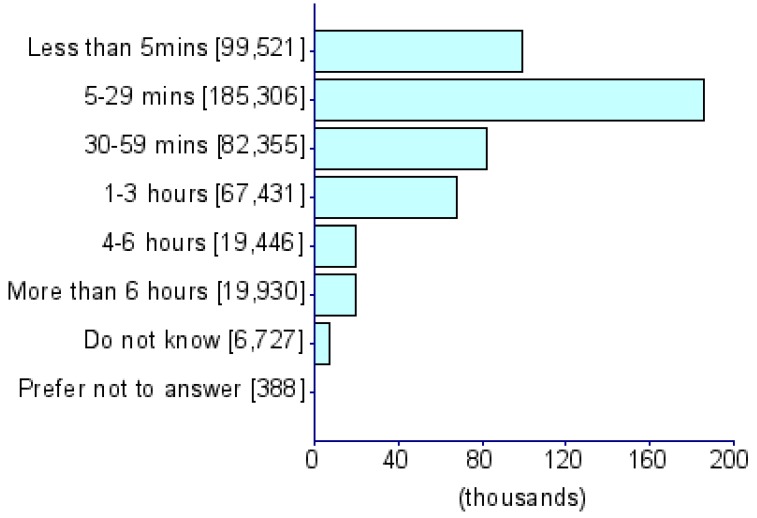
Hands-free device/speakerphone use with mobile phone in last 3 months (UKB ID: 1130)	*Over the last three months, how often have you used a hands-free device/speakerphone when making or receiving calls on your mobile?*	*HandsFree*	302,733	7.15 (1.47)	UKB	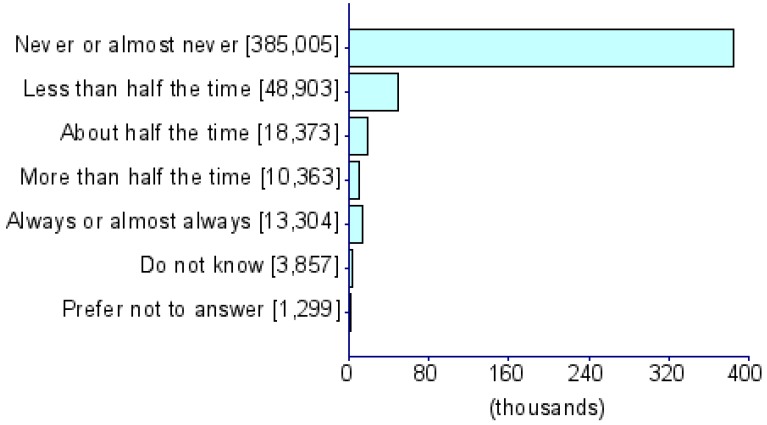
Differences in mobile phone use compared to two years previously (UKB ID: 1140)	*Is there any difference between your mobile phone use now compared to two years ago?*	*PhoneDifference*	298,239	5.44 (1.30)	UKB	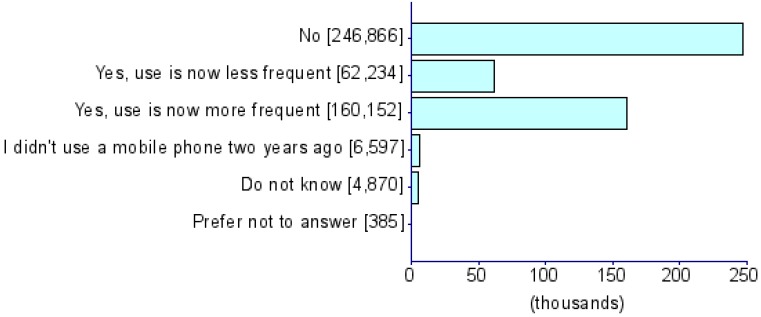
Plays computer games (UKB ID: 2237)	*Do you play computer games?*	*CompGaming*	360,817	7.28 (0.29)	UKB	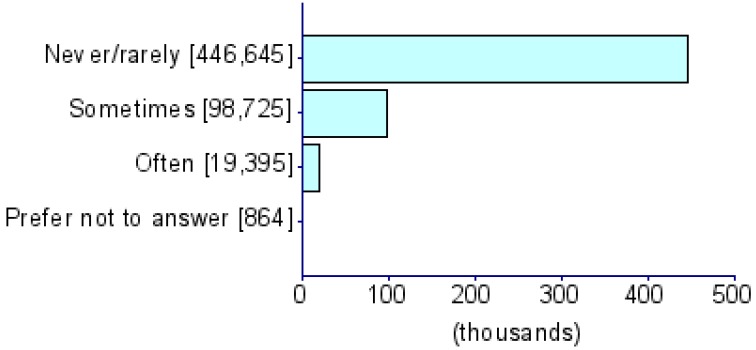
Attention Deficit Hyperactivity Disorder	-	ADHD	19,099 cases, 34,194 controls	22.81 (1.48)	PGC/iPSYCH	[17]
Alcohol Dependence	-	AD	8,485 cases, 23,080 controls	5.08 (1.16)	PGC	[18]
Autism Spectrum Disorder	-	ASD	18,382 cases, 27,969 controls	19.41 (1.68)	PGC/iPSYCH	[20]
Schizophrenia	-	SCZ	36,989 cases, 113,075 controls	23.22 (0.92)	PGC	[24]
Post-traumatic Stress Disorder	-	PTSD	13,638 cases, 15,548 controls	4.86 (2.15)	PGC	[23]
Anorexia Nervosa	-	AN	3,495 cases, 10,982 controls	24.03 (3.82)	PGC	[19]
Major Depressive Disorder	-	MDD	59,851 cases, 113,154 controls	7.61 (0.44)	PGC	[22]
Bipolar Disorder	-	BIP	20,352 cases, 31,358 controls	4.32 (12)	PGC	[21]

## Supplementary Materials

The following are available online at https://www.mdpi.com/2077-0383/8/12/2040/s1, Figure S1: Causal estimates, Figure S2: Multivariable causal estimates for schizophrenia and *CompGaming*, Figure S3: Multivariable causal estimates for ADHD and *PhoneUse*, Figure S4: Analytic pipeline, Table S1: Genetic correlations between CDU and psychiatric disorders, Table S2: Mendelian randomization estimates and sensitivity tests, Table S3: Multivariable Mendelian randomization results, Table S4: Latent causal variable results, Table S5: Genomic risk loci for schizophrenia, Table S6: Genomic risk loci for *CompGaming*, Table S7: Gene set enrichment differences schizophrenia and *CompGaming*, Table S8: Schizophrenia gene sets, Table S9: *CompGaming* gene sets, Table S10: Genetic correlations shared between schizophrenia and *CompGaming*, Table S11: Genomic risk loci for ADHD, Table S12: Genomic risk loci for *PhoneUse*, Table S13: Genomic risk loci for autism spectrum disorder, Table S14: Genomic risk loci for ADHD in females only, Table S15: Genomic risk loci for ADHD in males only, Table S16: Genomic risk loci *PhoneUse* in females only, Table S17: Genomic risk loci for *PhoneUse* in males only, Table S18: Gene sets shared between ADHD and *PhoneUse*, Table S19: Gene sets shared between ADHD and *PhoneUse* for females only, Table S20: Gene sets shared between ADHD and *PhoneUse* for males only, Table S21: Genetic correlations shared between ADHD and *PhoneUse*, Table S22: Genetic correlations shared between ADHD and *PhoneUse* for females only, Table S23: Genetic correlations shared between ADHD and *PhoneUse* in males only, Table S24: Gene set enrichment differences between autism spectrum disorder and *PhoneUse*, Table S25: Genetic correlations shared between autism spectrum disorder and *PhoneUse*, Additional Data Table 1: Genetic instruments for schizophrenia and CompGaming, Additional Data Table 2: Genetic instruments for ADHD and *PhoneUse*, Additional Data Table 3: Genetic instruments for autism spectrum disorder and *PhoneUse*.
